# Liver transcriptome resources of four commercially exploited teleost species

**DOI:** 10.1038/s41597-020-0565-9

**Published:** 2020-07-07

**Authors:** André M. Machado, Antonio Muñoz-Merida, Elza Fonseca, Ana Veríssimo, Rui Pinto, Mónica Felício, Rute R. da Fonseca, Elsa Froufe, L. Filipe C. Castro

**Affiliations:** 1 CIIMAR - Interdisciplinary Centre of Marine and Environmental Research, U. Porto – University of Porto, Porto, Portugal; 2 CIBIO-InBIO, Research Network in Biodiversity and Evolutionary Biology, Universidade do Porto, Campus Agrário de Vairão, 4485-661 Vairão, Portugal; 3 Department of Biology, Faculty of Sciences, U. Porto - University of Porto, Porto, Portugal; 4 Portuguese Institute for the Sea and Atmosphere, I.P. (IPMA), Lisbon, Portugal; 5 Center for Macroecology, Evolution and Climate, Natural History Museum of Denmark, University of Copenhagen, Copenhagen, Denmark

**Keywords:** Transcriptomics, Ecological genetics

## Abstract

The generation of *omic* resources is central to develop adequate management strategies for species with economic value. Here, we provide high-coverage RNA-seq datasets of liver tissue (containing between 80,2 and 88,4 million of paired-end reads) from four wildtype teleost species with high commercial value: *Trachurus trachurus* (TTR; Atlantic horse mackerel), *Scomber scombrus* (SSC; Atlantic mackerel), *Trisopterus luscus* (TLU; pout), and *Micromesistius poutassou* (MPO; blue whiting). A comprehensive assembly pipeline, using *de novo* single and multi-kmer assembly approaches, produced 64 single high-quality liver transcriptomes – 16 per species. The final assemblies, with N50 values ranging from 2,543–3,700 bp and BUSCO (Benchmarking Universal Single-Copy Orthologs) completeness values between 81.8–86.5% of the Actinopterygii gene set, were subjected to open reading frame (ORF) prediction and functional annotation. Our study provides the first transcriptomic resources for these species and offers valuable tools to evaluate both neutral and selected genetic variation among populations, and to identify candidate genes for environmental adaptation assisting in the investigation of the effects of global changes in fisheries.

## Abstract (summary)

**Table:** 

Measurement(s)	RNA • transcriptome • sequence_assembly • sequence feature annotation
Technology Type(s)	RNA sequencing • sequence assembly process • sequence annotation
Factor Type(s)	teleost species
Sample Characteristic - Organism	Trachurus trachurus • Scomber scombrus • Trisopterus luscus • Micromesistius poutassou

Machine-accessible metadata file describing the reported data: 10.6084/m9.figshare.12533171

## Background & Summary

Multi-data approaches using complementary techniques are essential to successfully define fish stocks and management strategies (e.g.^1,2^). The revolution of Next-Generation Sequencing (NGS) has created an unprecedented opportunity to contribute to each component of fisheries management (e.g. reviewed in^3^), allowing to address population structure and adaptive divergence in commercially relevant teleost fish species (e.g. Atlantic cod^4^ and Atlantic herring^5^), to identify candidate genes for environmental adaptation^3^ or to explore the function of genes with aquaculture relevance^6^. Importantly, the expansion of genomic and transcriptomic datasets has been fundamental to detail the complex phylogenetic relationships of this taxon-rich clade^7–9^. Additionally, these resources have also been proven important in conservation strategies, where they have allowed the prediction of how species will respond to new environmental scenarios and the identification of the threats endangering species at risk (e.g.^10^). Yet, these formidable tools have to be applied to the vast majority of the world fisheries: 60% of which are at their maximum sustainable yield, 33% are exploited at biologically unsustainable levels and only 7.0% are considered underfished^11^.

**Fig. 1:**
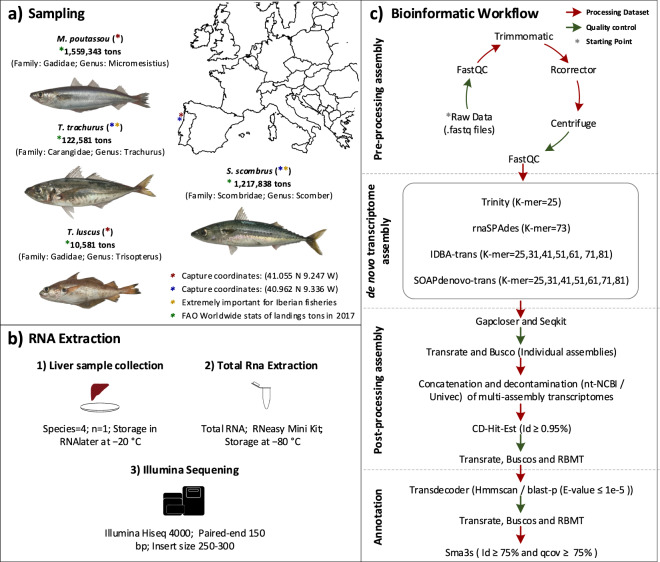
Schematic overview of the study. (**a**) Geographic coordinates of sampling, photographs of the collected specimens, taxonomy classification and fishery relevance. (**b**) Experimental setup used to perform RNA extraction and sequencing. (**c**) Bioinformatics workflow used to post-sequencing dataset analyses.

Here, we generated four liver transcriptomic datasets from important fishing resources in European waters belonging to three different taxonomic families: the Atlantic horse mackerel, *Trachurus trachurus* (Linnaeus 1758), the Atlantic mackerel, *Scomber scombrus* (Linnaeus 1758) (SSC), the pout, *Trisopterus luscus* (Linnaeus 1758) and the blue whiting, *Micromesistius poutassou* (Risso 1827) (Fig. 1a). Together, these species represent an important fraction of fish captures in European waters (Fig. 1a) and are, therefore, relevant models for which the development of *omic* tools for research is highly desirable. Although some information regarding basic biological traits is currently available, some aspects of their biology remain poorly known, particularly population structure, nutritional requirements, reproduction, coastal recruitment processes, and studies involving NGS datasets are also scarce or absent. For example, in *Scomber scombrus* two RNA-seq projects are available (PRJNA272777 (low coverage RNA-seq dataset produced from a pool of tissues (muscle, liver, gonad, brain) and 454 GS-FLX Titanium System) and PRJNA305977 (RNA-seq dataset produced from white muscle tissue and with Illumina HiSeq2000 system))^12,13^. For the remaining species, no RNA-seq data is currently available. To cover the above-mentioned biological aspects, the liver was selected to produce a high-quality and coverage transcriptome for each species. Tipically, this tissue has a large number of expressed genes. On the other hand, the selection of a single specimen per species avoided the intraspecies variations, which associated with heterozygosity levels of marine fish species, generally higher than freshwater species, makes the construction of high-quality genomic and transcriptomic references a complex and challenging task.

## Methods

### Animal sampling

Adult specimens of *T. trachurus, S. scombrus*, *T. luscus, and M. poutassou* were collected to perform the RNA-seq analyses. Individuals were caught in the Northeast Atlantic Ocean, Portugal: *T. trachurus* and *S. scombrus* (40.961667 N, 9.336000 W) and *T. luscus and M. poutassou* (41.055000 N, 9.246667 W), under the guidelines of the “*Programa Nacional de Amostragem Biológica*”, conducted by the Instituto Português do Mar e da Atmosfera (IPMA) (Fig. 1a; Table 1). Immediately upon capture, liver tissue from each specimen was collected and stored in RNAlater RNA Stabilization Reagent (Qiagen, Germany) during 24 h at 4 °C (Fig. 1b). The samples were later transferred to −80 °C until total RNA extraction (Fig. 1b).

**Table 1:** MixS descriptors of four commercial fish species used on this study.

Species	*T. trachurus*	*S. scombrus*	*T. luscus*	*M. poutassou*
Investigation_type	Eukaryote	Eukaryote	Eukaryote	Eukaryote
Project_name	Liver transcriptome of four commercial fish species			
Lat_lon	40.961667 N 9.336000 W	40.961667 N 9.336000 W	41.055000 N 9.246667 W	41.055000 N 9.246667 W
Geo_loc_name	NorthEast Atlantic Ocean	NorthEast Atlantic Ocean	NorthEast Atlantic Ocean	NorthEast Atlantic Ocean
Collection_date	6/22/2017	6/22/2017	6/22/2017	6/22/2017
Biome	Coastal sea water (ENVO:00002150)	Coastal sea water (ENVO:00002150)	Coastal sea water (ENVO:00002150)	Coastal sea water (ENVO:00002150)
Feature	Coastal water body (ENVO:02000049)	Coastal water body (ENVO:02000049)	Coastal water body (ENVO:02000049)	Coastal water body (ENVO:02000049)
Material	Sea water (ENVO:00002150)	Sea water (ENVO:00002150)	Sea water (ENVO:00002150)	Sea water (ENVO:00002150)
Env_package	Water	Water	Water	Water
Seq_meth	Illumina HiSeq4000	Illumina HiSeq4000	Illumina HiSeq4000	Illumina HiSeq4000
Assembly method	Multiple Methods (Trinity, rnaSPAdes, SOAPdenovo-trans, IDBA-trans)	Multiple Methods (Trinity, rnaSPAdes, SOAPdenovo-trans, IDBA-trans)	Multiple Methods (Trinity, rnaSPAdes, SOAPdenovo-trans, IDBA-trans)	Multiple Methods (Trinity, rnaSPAdes, SOAPdenovo-trans, IDBA-trans)
Collector	Mónica Felicio	Mónica Felicio	Mónica Felicio	Mónica Felicio
Sex	female	female	male	female
Fork length	28,3 cm	40,5 cm	18 cm	21,5 cm
Maturity	Mature	Mature	Mature	Mature

### RNA extraction, library construction, and sequencing

Total RNA was extracted from liver using the Illustra RNAspin Mini RNA Isolation Kit (GE Healthcare, UK), according to the manufacturer’s instructions. The isolated RNA was treated with RNase-free DNase I to remove residual genomic DNA contamination and eluted in RNase-free water. RNA concentration was measured using a microplate spectrophotometer with Take3™ Micro-Volume Plate (BioTeK, USA) (*T. trachurus* - 2816,556 ng/μl, *S. scombrus -* 2379,382 ng/μl, *T. luscus -* 1147.368 ng/μl, *and M. poutassou -* 1236.980 ng/μl). The RNA quality was verified with the measurement of the OD260/280 ratio values (1.8 to 2.0). The integrity of each RNA sample was checked by running 1 μl in a 1% agarose gel. Afterwards, the four RNA samples were used to build four strand-specific libraries, one per species, with an insert size of 250–300 bp and sequenced using 150 bp paired-end reads on the Illumina HiSeq4000 platform by Novogene (China).

### Pre-assembly processing stage

The raw dataset for each specimen was initially inspected with the FastQC (version 0.11.8) software (http://www.bioinformatics.babraham.ac.uk/projects/fastqc/). Trimmomatic (version 0.38)^14^ was then used to trim, quality-filter the raw reads and remove Illumina adaptors, under the following parameters (LEADING:5 TRAILING:5 SLIDINGWINDOW:5:20 MINLEN:36) (Fig. 1c). To correct random sequencing errors introduced during the sequencing or in another stage of the pre-*in silico* processing, we applied a kmer-based error correction method, Rcorrector (version 1.0.3)^15^, with default settings. At this stage, all the unfixable reads were discarded. The error-corrected reads were posteriorly introduced in the Centrifuge (version 1.0.3-beta)^16^ program and taxonomically classified against the pre-compiled nucleotide database of NCBI (ftp://ftp.ccb.jhu.edu/pub/infphilo/centrifuge/data/) (version nt_2018_3_3). Importantly, all the reads not classified as belonging to Actinopterygii superclass (Taxon Id: 7898) were considered exogenous to our target species and removed from the initial datasets (Fig. 1c).

### *De novo* transcriptome assembly stage

To build the transcriptome, we opted by the *de novo* assembly strategy using a multi-kmer approach. Thus, to generate the liver transcriptomes of TTR, SSC, TLU, and MPO we used four assemblers – Trinity (version 2.8.4)^17,18^, rnaSPAdes mode of SPAdes (version 3.11.1)^19^, SOAPdenovo-trans (version 1.03)^20^, and IDBA-trans (version 1.1.3)^21^ (Fig. 1c). The first assembly was carried out by Trinity software with a fixed Kmer of 25 and a strand-specific data parameter on (SS_lib_type RF). The RnaSPAdes tool was then applied with the default parameters (kmer of 73, following the strategy used in the original publication (read length/2−1)^19^. In the remaining assemblers, we used a multi-kmer approach that required both the mean insert size (IS) and standard deviation (SD) values of the raw dataset. To calculate these values, we used the transcriptome generated by Trinity as a reference, the Bowtie2 (version 2.3.5)^22^ to map the clean raw reads, and finally CollectInsertSizeMetrics function of Picard tools (version 2.19.2)^23^ to estimate the insert size and standard deviation values. The IDBA-trans assemblies were performed with the SD and IS, previously calculated, the kmer values of 25, 31, 41, 51, 61, 71, 81 and the–ss-fr parameter on. On the other hand, SOAPdenovo-trans used the kmer values of 25, 31, 41, 51, 61, 71, 81 with (-L 200; -F YES) parameters. To remove the gaps inserted during the SOAPdenovo-trans assemblies, the GapCloser (version 1.12) module of SOAPdeonovo2^24^ software was used, with default settings.

### Post-assembly processing stage

In the post-assembly processing stage, all assemblies were processed with the SeqKit (version 0.10.1) toolkit^25^. This tool removed all contigs with less than 200 nucleotides, and concatenated all assemblies, per species, in one multi-assembly file. Furthermore, we also conducted several searches against the nucleotide NCBI (nt-NCBI) (downloaded on 30/03/2019) and UniVec (downloaded on 02/04/2019) databases to identify and remove biological contaminations, vectors or adapters not identified in the previous stages. These searches were done via blast-n (version 2.9.0) against the nt-NCBI database with the parameters (-evalue 1e-5; -max_target_seqs. 1; -perc_identity 90; -max_hsps 1; and minimum alignment length of 50 bp), and against UniVec database with the settings (-reward 1; -penalty -5; -gapopen 3; -gapextend 3; -dust yes; -soft_masking true; -evalue 700; -searchsp 1750000000000). For the nt-NCBI searches, all contigs with the best match hits out of the Actinopterygii taxon were considered contaminations and removed from the transcriptome assemblies. The remaining transcripts, without any match hit or with match hits in Actinopterygii taxon were kept into the transcriptomes and used for further analyses. Regarding the Univec database, all transcripts with a match hit were considered exogenous and removed from the dataset. To decrease the redundancy and complexity within the decontaminated transcriptomes, we clustered highly similar nucleotide sequences with the CD-HIT-EST (version 4.7)^26^ software, with the following settings (-c 0.95; -g 1; -M 60000; -T 30) (Fig. 1c). Essentially, the software clusters and compares nucleotide sequences, keeping the longest sequence per cluster above a certain similarity threshold, in our case 95% of similarity. All steps of the transcriptome assembly and post-processing stage (from the single kmer assemblies build until the concatenation, decontamination and the clean-up of redundancy) were further inspected to guarantee the accuracy of our approach. Thus, we used the Trinity and Transrate (1.0.3)^27^ for primary statistics and the Benchmarking Universal Single‐Copy Orthologs (BUSCO version 3.0.2)^28^ – with four lineage-specific profile libraries (Metazoa, Eukaryota, Vertebrates and Actinopterygii) – to evaluate the gene completeness of each assembly. In addition, the rate of reads back mapping to the transcriptome (RBMT) was also calculated for all the assemblies after the decontamination step (Fig. 1c). The RBMT was performed with Bowtie2 (version 2.3.5)^22^ tool.

### Open reading frame prediction and transcriptome annotation

The open reading frames (ORFs) were predicted using the Transdecoder software (version 5.3.0) (https://transdecoder.github.io/) (Fig. 1c). This pipeline is mainly subdivided into three stages. In the first stage, the software pre-predicted the longest ORF per transcript with a cut off length of 100 aminoacids. In the second stage, to find homology and protein evidence, two databases were screened – blast-p (version 2.9.0) with cut-off evalue of 1e-5 against UniProtKB/Swiss-Prot database (downloaded on 12/04/2019)^29^ and hmmscan of hmmer2 package (version 2.4i)^30^ to find protein profiles against PFAM database (downloaded on 12/04/2019)^31^, respectively. In the last stage, all the information collected from both databases, together with the pre-predicted ORF’s were used to perform the final prediction of the amino acid sequence. Afterwards, all transcripts codifying for a protein, per species, were used to carry out a functional annotation step with the Sma3s (version 2.1)^32^ tool (Fig. 1c). Functional annotation was assigned applying consecutive filters to a blast record (performed against the Uniref90 database (downloaded on 2019-02)) based on both similarity and query coverage. Functional domains were also identified clustering all significant blast hits, and their annotations were retrieved only when their frequency was higher than the frequency of appearance in the reference database following the hypergeometric distribution. Annotation types retrieved consist of GO terms, EC codes from ENZYME repository and Uniprot keywords and pathways. Gene name is only associated in cases of a blast hit greater than 75% of identity and 75% of query coverage.

## Data Records

The data generated in this study is subdivided in three main categories: the raw reads, the transcriptome assemblies, and the functional annotation. The decontaminated raw reads, for each species, were deposited in the NCBI Sequence Read Archive – SRP216187^33^. Furthermore, the non-redundant transcriptome assemblies were submitted in fasta format, to the NCBI Transcriptome Shotgun Assembly Sequence Database, under the accession number, GHRS00000000, (TTR), GHRT00000000 (SSC), GHRZ00000000 (TLU), GHRY00000000 (MPO)^34–37^. The remaining transcriptome assemblies (fasta format), the open reading frames, as well the annotation files per species were uploaded to figshare online repository^38^. In detail, it is possible to consult in figshare, the 16 initial individual transcriptome assemblies of the four species produced with four different assemblers, the multi assembly and the final assembly, per species, in fasta format. In addition, also the predicted open reading frames (.pep file), as well as the remaining outputs of TransDecoder software (.bed,.gff3 and.cds files) and the functional annotation files are available, per species^38^.

## Technical Validation

### Raw datasets and pre-assembly processing quality control

**Fig. 2:**
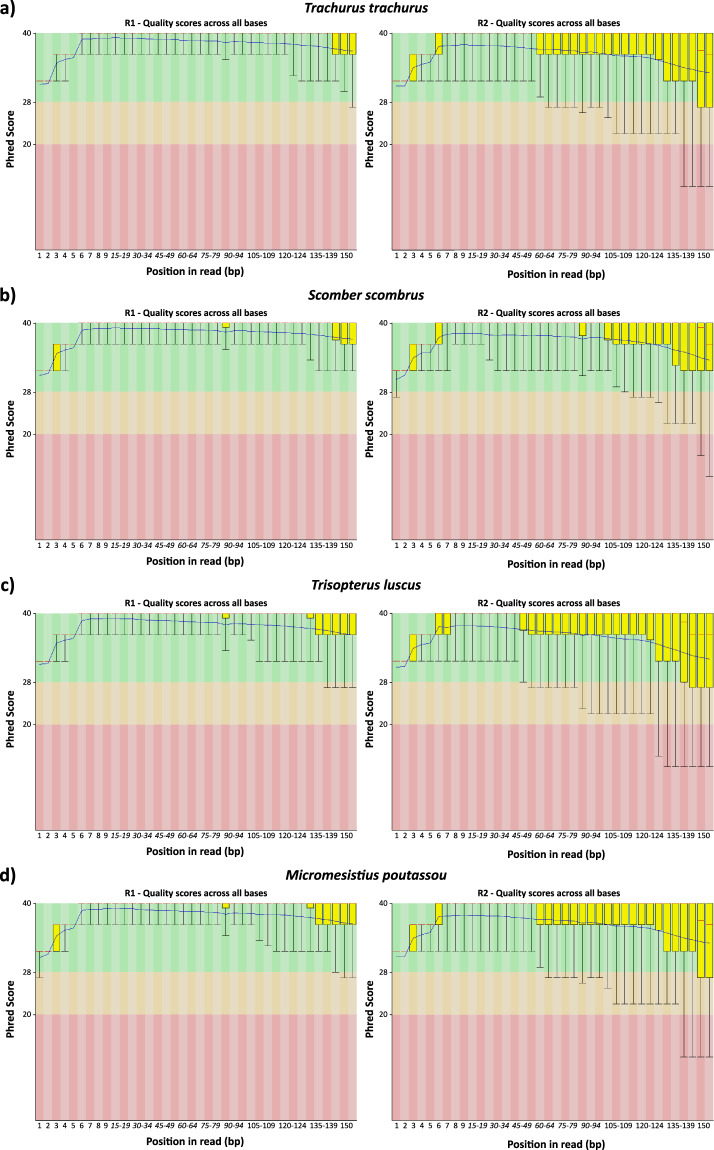
Quality FastQC report of post-processing RNA-seq datasets (after Centrifuge (version 1.0.3-beta)^16^ cleaning stage). For each species, it is presented the R1 and R2 FastQC reports. (**a**) *Trachurus trachurus*; (**b**) *Scomber scombrus*; (**c**) *Trisopterus luscu*; (**d**) *Micromesistius poutassou*.

The sequencing process generated a total of 88,4 M in TTR, 87,8 M in SSC, 80,2 M in TLU and 84 M in MPO paired-end raw reads. All raw datasets were initially scrutinized by the FastQC tool, trimmed with Trimmomatic, error-corrected with Rcorrector and decontaminated with Centrifuge software. The percentage of removed reads per step in the pre-assembly processing stage can be visualized in Table 2. Overall, ~86,7 M in TTR, 87,1 in SSC, 79,2 in TLU and 82,8 MPO reads had Phred scores higher than 20 and were retained for the transcriptome assembly stage (Table 2 and Fig. 2a–d ^33^).

**Table 2:** Technical features of raw datasets and percentages of raw reads removed in each step of the pre-assembly processing stage.

Raw Reads	*T. trachurus*	*S. scombrus*	*T. luscus*	*M. poutassou*
Raw sequencing reads	88451325	87805244	80273856	84099678
Trimmomatic reads removed	62084 (0.07%)	43757 (0.05%)	49140 (0.06%)	63070 (0.07%)
Centrifuge reads removed	1658993 (1.88%)	585774 (0.67%)	965820 (1.20%)	1145446 (1.36%)
Reads used in assembly	86730248 (98.05%)	87175713 (99.29%)	79258896 (98.74%)	82891162 (98.52%)
**Technical features**	—			
Median Insert size	274	264	277	275
Mode insert size	266	262	271	268
Median Absolute Deviation	40	39	41	40
Minimum Insert size	122	124	123	122
Maximo Insert size	887	889	1013	1812
Mean insert size	277.884336	267.483079	280.906177	278.967563
Standard Deviation	61.001219	59.050183	61.544275	60.196021

### Transcriptome assembly metrics

The *de novo* transcriptome assembly was performed using multiple software including Trinity, rnaSPAdes, SOAPdenovo-trans and IDBA-trans. This strategy has been previously applied with success in transcriptome projects of multiple organisms, without a reference genome^39–41^. The first assembly obtained with Trinity tool was used to map the raw decontaminated reads and to calculate the IS and SD for each species. In all species, the IS and SD values ranged between 267–280 and 59–61 (Table 2). The initial multi-assembly approach generated 64 transcriptome assemblies, 16 for each species^38^. The statistic metrics for each assembly, such as N50 transcript length, mean transcript length, percentage of GC, longest transcript length, or transcript number over 1k or 10k nucleotides can be visualized in Online-only Tables 1–4. In addition, we also performed BUSCO analyses using 303, 978, 2586 and 4584 near-universal single-copy orthologs in Eukaryota, Metazoa, Vertebrata, and Actinopterygii gene sets, respectively, for all the 16 assemblies (Online-only Tables 1–4). Regarding the individual assemblies, the Trinity tool presented the higher values of N50 and mean transcript length in all species: TTR – 1708 and 921,55 bp, SSC – 1964 and 984,48 bp, TLU – 1321 and 780,05 bp, and MPO – 1228 and 782.03 bp, respectively (Online-only Tables 1–4). Moreover, the BUSCO analyses revealed the same pattern with higher percentage of total genes found (complete + fragmented) in Trinity assemblies for all lineage-specific profile libraries consulted (Eukaryota, Metazoa, Vertebrata and Actinopterygii): TTR – 100.00, 99.28, 90.37, 81.72%, SSC – 99.01, 99.28, 89.29, 79.89%, TLU – 100.00, 99.80, 92.34, 83.40%, and MPO – 100.00, 99.69, 92.42, 83.99% (Online-only Tables 1–4). The magnitude of these values is comparable and, in some cases, superior to several fish transcriptomes, publicly available, e.g. (e.g. *Xiphias gladius –* 99 and 98.2% of genes found in Eukaryota, Metazoa databases^42^; *Amphiprion percula –* 85.4% of genes found in Actinopterygii database^43^), which suggests a high quality of our initial assemblies.

### Post-assembly processing and annotation verification

At this stage, the 16 assemblies per species were concatenated and decontaminated, resulting in four multi-assembly transcriptomes – Online-only Table 4 ^38^. During the decontamination phase, all blast-n results were manually inspected and the threshold of minimum alignment length of 50 bp and taxonomic superclass Actinopterygii, were specifically selected due to two factors: the considerable number of plausible biological contamination hits with more 50 bp of nucleotide alignment length (e.g. *Lasthenia californica* (Taxon ID: 149440)) and the relatively high number of Actinopterygii species with genome sequenced and annotated on nt-NCBI database (at least 43 species), respectively. Although, some transcripts have been discarded at this stage (e.g. novel sequences not present in this 43 Actinopterygii species), we have ensured the application of sensitive blast parameters. Thus, instead of focusing the analyses on the removal of all sequences with a match hit, we performed first an effort on the identification of the hits, *via* homology, against the possible sources of contamination and only after excluded if confirmed. Apart from a few transcripts that have been removed, this approach increased the confidence levels of each dataset. Posteriorly, four multi-assemblies were subject to a redundancy removal step to decrease the complexity and to remove the overlapping transcripts above 95% of sequence similarity^34–37^. This method has been highly used to remove redundancy in several datasets and organisms^40,44–46^, namely in the build of new transcriptomic references. In addition, this strategy still decreased the natural heterozygoty of the organisms in the assemblies. It should be noted that although naturally present in the organisms, the heterozygosity continues to be a crucial parameter to consider during the generation of transcriptomic and genomic datasets. In some cases, where heterozygosity is not contemplated in the bioinformatic approach, the results change completely and their interpretation can be affected. Using this approach, the total number of transcripts in each dataset was substantially reduced, TTR – 2769441 to 414729, SSC – 2728965 to 377586, TLU – 3203445 to 548983, and MPO – 3675167 to 602418, allowing a better overview and understanding of the datasets (Online-only Table 5). After this step, we implemented another quality control strategy, the RBMT. This method, together with the already established metrics, general statistics, and gene completeness, allowed us to verify the raw read content of the transcriptomes. Importantly, this metric showed that even with the redundancy removal step, the RBMT rate in the non-redundant transcriptomes has kept very high values, TTR – 96.78, SSC – 97.16, TLU – 95.3, MPO – 95.51% (Online-only Table 5).

In the process of ORF prediction, the TransDecoder software identified TTR – 111866, SSC – 97811, TLU – 150334, MPO – 167124 transcripts with an assigned ORF. Importantly, the entire set of transcripts codifying for a protein was collected from the non-redundant transcriptomes and placed in files classified as final transcriptome assemblies^38^.

The basic metrics, BUSCO analyses and RBMT rates for TTR, SSC, TLU, and MPO final transcriptome assemblies are available in Table 3. Notwithstanding, the final transcriptome statistics demonstrate the power of the assembly and processing strategy chosen, with N50 values ranging from 2543 to 3700 bp, BUSCO values between 81.8–86.5% in Actinopterygii gene set, and raw reads rate mapping ranging from 91.45 to 94.63% (Table 3). Interestingly, the BUSCO analyses in Vertebrata and Actinopterygii gene sets still shown a decrease in the percentage of fragmented genes (less than half) and missing genes (slightly), in the final transcriptome assemblies when compared with the initial individual assemblies, for all species.

**Table 3:** Transrate, Busco and RBMT statistics of the final liver transcriptome assemblies of *T. trachurus*, *S. scombrus*, *T. luscus*, *M. poutassou*.

Basic statistics	*T. trachurus*	*S. scombrus*	*T. luscus*	*M. poutassou*
Number of transcripts	111866	97811	150334	167124
Longest transcript	29599	44312	32751	26373
n bases	223085715	237628172	247718527	288345963
Mean transcript lenght (bp)	1994.22	2429.46	1647.79	1725.34
Number of transcripts over 1 K nt	70814	68168	81638	92951
Number of transcripts over 10 K nt	567	1050	379	528
N90 trancript lenght (bp)	957	1212	739	776
N70 trancript lenght (bp)	1994	2477	1648	1739
N50 trancript lenght (bp)	2991	3700	2543	2699
N30 trancript lenght (bp)	4370	5249	3718	3926
N10 trancript lenght (bp)	7131	8290	6125	6431
Percentage of GC (%)	0.49	0.46	0.55	0.53
RBMT (%)	93.78	94.63	91.45	91.52
**Busco analysis (%)**	—			
BUSCO Complete (Single + Duplicated)*	99.01\97.85\83.99\78.42	97.69\97.44\84.49\76.81	99.01\98.47\88.05\81.85	99.01\98.88\88.90\82.42
BUSCO Single*	36.63\34.87\32.95\30.61	33.33\32.72\33.64\31.87	23.76\28.12\30.12\28.49	23.76\28.12\28.54\28.29
BUSCO Duplicated*	62.38\62.99\51.04\47.82	64.36\64.72\50.85\44.94	75.25\70.35\57.93\53.36	75.25\70.76\60.36\54.12
BUSCO Fragmented*	0.66\1.12\7.54\6.06	0.99\1.23\5.96\5.02	0.00\0.51\5.07\4.45	0.33\0.31\4.60\4.08
BUSCO Missing*	0.33\1.02\8.47\15.51	1.32\1.33\9.55\18.17	0.99\1.02\6.88\13.70	0.66\0.82\6.50\13.50
Total Buscos Found*	99.67\98.98\91.53\84.49	98.68\98.67\90.45\81.83	99.01\98.98\93.12\86.30	99.34\99.18\93.50\86.50
**Annotation**	—			
Transcripts with ORF	111866	97811	150334	167124
Transcrips annotated with Gene Name	87847	77369	93067	102433
Transcrips annotated with GO terms	88269	78116	93274	104086
Transcrips annotated with ENZYME	32485	29241	34832	39244
Transcrips annotated with PATHWAY	8653	7462	8712	9760
Final number of transcrips annotated	90428	79911	95110	106354

*euk/met/ver/act.

Euk: Dataset with 303 genes of Eukaryota library profile.

Met: Dataset with 978 genes of Metazoa library profile.

Ver: Dataset with 2586 genes of Vertebrata library profile

Actino: Dataset with 4584 genes of Actinopterygii library profile.

In the end, the final transcriptomes were functionally annotated using the Sma3s software. A high number of the transcripts were annotated and most of them including the gene name which suggests a remarkable quality of the assemblies. Annotation distribution across the different species is very similar, keeping a logical proportion based on the total number of transcripts. All the annotations stats, including the gene homology, the most probable gene name, the GO terms, Kegg Pathways and EC numbers for Enzymes can be consulted in Table 3 ^38^.

## Data Availability

All the software programs used in the bioinformatics workflow (transcriptome assembly, pre and post-assembly processing stages and transcriptome annotation) are presented in the Methods section. All programs and databases have the versions, download dates, and parameters described. Software programs with no parameters associated were used with the default settings.

## Online-only Tables

**Online-only Table 1:** Transrate and Busco statistics of the 16 initial liver transcriptome assemblies of T. trachurus.

Basic statistics	Trinity	RnaSpades	SOAPdenovo-Trans (k25)	SOAPdenovo-Trans (k31)	SOAPdenovo-Trans (k41)	SOAPdenovo-Trans (k51)	SOAPdenovo-Trans (k61)	SOAPdenovo-Trans (k71)	SOAPdenovo-Trans (k81)	Idba-trans (k25)	Idba-trans (k31)	Idba-trans (k41)	Idba-trans (k51)	Idba-trans (k61)	Idba-trans (k71)	Idba-trans (k81)
Number of transcripts	335497	245191	125230	131782	144544	159289	175732	174999	151673	145474	145126	152429	161206	171675	179629	183528
Longest transcript	29599	20910	19537	20428	18720	17339	17828	19243	16992	16618	17032	19198	15600	14635	13980	12397
n bases	309175642	177721497	89820450	92779476	95333230	97574502	100078540	99338997	93415859	80564095	84494772	85909498	87246182	88331398	88582303	87650963
Mean transcript lenght (bp)	921.55	724.83	717.24	704.04	659.54	612.56	569.5	567.65	615.9	553.8	582.22	563.6	541.21	514.53	493.14	477.59
Number of transcripts over 1 K nt	84012	41005	21399	22015	22015	22130	22218	22451	22506	16562	18560	18064	17605	16568	15662	14489
Number of transcripts over 10 K nt	403	191	109	110	93	82	49	48	29	9	14	15	16	10	7	11
N90 trancript lenght (bp)	346	275	267	262	249	239	232	237	258	264	267	262	257	250	243	238
N70 trancript lenght (bp)	819	517	570	553	503	450	401	394	444	421	442	421	398	373	357	347
N50 trancript lenght (bp)	1708	1382	1274	1238	1116	997	888	865	949	679	742	707	664	610	564	534
N30 trancript lenght (bp)	2930	2757	2514	2466	2287	2047	1821	1732	1803	1135	1291	1247	1191	1109	1033	965
N10 trancript lenght (bp)	5445	5512	5167	5139	4780	4448	3922	3645	3604	2262	2641	2631	2565	2434	2306	2220
Percentage of GC (%)	0.47	0.46	0.46	0.46	0.46	0.46	0.46	0.47	0.47	0.46	0.46	0.46	0.46	0.46	0.46	0.47
**Busco analysis (%)**	—															
BUSCO Complete (Single + Duplicated)*	97.36\96.83\78.27\71.97	95.38\95.91\75.33\67.69	97.03\95.40\78.27\70.40	96.37\94.79\76.91\69.48	91.42\92.74\73.32\67.15	91.09\91.72\69.88\63.26	85.81\87.22\62.34\56.98	84.82\83.74\57.15\52.86	79.87\83.13\55.96\49.80	65.02\66.87\41.11\33.92	67.66\69.02\42.54\34.97	69.64\71.88\41.69\35.23	74.26\76.48\46.67\39.86	77.23\78.94\47.22\40.64	79.87\80.88\48.41\42.41	78.22\81.70\49.54\42.52
BUSCO Single*	13.53\10.33\13.19\12.91	69.31\67.38\55.30\49.19	89.77\89.06\74.05\65.53	89.77\87.32\72.27\64.31	84.82\85.99\68.21\62.00	84.49\82.31\64.58\57.11	77.89\76.89\55.45\49.85	73.93\70.55\49.19\44.31	69.31\67.59\45.82\40.07	63.04\64.31\40.29\33.25	64.69\66.26\41.61\33.97	66.34\69.02\40.95\34.40	70.96\72.49\45.82\38.66	73.93\74.34\46.25\39.40	77.23\76.58\47.37\41.08	76.90\77.20\48.41\41.30
BUSCO Duplicated*	83.83\86.50\65.08\59.05	26.07\28.53\20.03\18.50	7.26\6.34\4.22\4.86	6.60\7.46\4.64\5.17	6.60\6.75\5.10\5.15	6.60\9.41\5.30\6.15	7.92\10.33\6.88\7.13	10.89\13.19\7.97\8.55	10.56\15.54\10.13\9.73	1.98\2.56\0.81\0.68	2.97\2.76\0.93\1.00	3.30\2.86\0.73\0.83	3.30\3.99\0.85\1.20	3.30\4.60\0.97\1.24	2.64\4.29\1.04\1.33	1.32\4.50\1.12\1.22
BUSCO Fragmented*	2.64\2.45\12.10\9.75	4.62\3.68\15.58\13.31	2.97\3.68\12.14\11.26	3.30\4.09\12.99\11.63	7.59\6.03\15.55\12.67	7.26\6.44\18.41\14.86	12.54\10.94\24.48\18.26	13.53\14.42\28.38\20.20	17.49\14.83\28.42\21.14	31.02\28.73\42.19\29.73	29.70\28.32\41.34\31.06	27.06\25.36\42.27\30.78	23.10\21.37\38.05\28.21	21.12\19.02\37.24\27.51	18.48\17.69\36.43\26.42	19.80\16.67\34.15\25.00
BUSCO Missing*	0.00\0.72\9.63\18.28	0.00\0.41\9.09\19.00	0.00\0.92\9.59\18.35	0.33\1.12\10.09\18.89	0.99\1.23\11.14\20.18	1.65\1.84\11.72\21.88	1.65\1.84\13.19\24.76	1.65\1.84\14.46\26.94	2.64\2.04\15.62\29.06	3.96\4.40\16.71\36.34	2.64\2.66\16.13\33.97	3.30\2.76\16.05\33.99	2.64\2.15\15.27\31.94	1.65\2.04\15.55\31.85	1.65\1.43\15.16\31.17	1.98\1.64\16.32\32.48
Total Buscos Found*	100.00\99.28\90.37\81.72	100.00\99.59\90.91\81.00	100.00\99.08\90.41\81.65	99.67\98.88\89.91\81.11	99.01\98.77\88.86\79.82	98.35\98.16\88.28\78.12	98.35\98.16\86.81\75.24	98.35\98.16\85.54\73.06	97.36\97.96\84.38\70.94	96.04\95.60\83.29\63.66	97.36\97.34\83.87\66.03	96.70\97.24\83.95\66.01	97.36\97.85\84.73\68.06	98.35\97.96\84.45\68.15	98.35\98.57\84.84\68.83	98.02\98.36\83.68\67.52

*euk/met/ver/act

Euk: Dataset with 303 genes of Eukaryota library profile.

Met: Dataset with 978 genes of Metazoa library profile.

Ver: Dataset with 2586 genes of Vertebrata library profile

Actino: Dataset with 4584 genes of Actinopterygii library profile.

**Online-only Table 2:** Transrate and Busco statistics of the 16 initial liver transcriptome assemblies of S. scombrus.

Basic statistics	Trinity	RnaSpades	SOAPdenovo-Trans (k25)	SOAPdenovo-Trans (k31)	SOAPdenovo-Trans (k41)	SOAPdenovo-Trans (k51)	SOAPdenovo-Trans (k61)	SOAPdenovo-Trans (k71)	SOAPdenovo-Trans (k81)	Idba-trans (k25)	Idba-trans (k31)	Idba-trans (k41)	Idba-trans (k51)	Idba-trans (k61)	Idba-trans (k71)	Idba-trans (k81)
Number of transcripts	338127	232079	147977	150881	155364	161248	167990	157372	127912	158669	154159	156448	159617	162132	163995	164176
Longest transcript	44312	31456	24021	18568	28780	23475	22324	21718	25449	13982	15855	15411	22675	14708	17172	23031
n bases	332877922	177812586	110023758	112372948	111808036	110297592	107667878	101838468	91484534	93886615	97249133	97433311	96459740	94515627	92012738	88753661
Mean transcript lenght (bp)	984.48	766.17	743.52	744.78	719.65	684.02	640.92	647.12	715.21	591.71	630.84	622.78	604.32	582.95	561.07	540.6
Number of transcripts over 1 K nt	88423	38863	24989	25416	24927	24225	23147	22368	21502	20994	23006	22423	21489	20243	19132	17727
Number of transcripts over 10 K nt	566	306	183	186	206	180	143	122	98	10	24	22	21	25	29	23
N90 trancript lenght (bp)	356	279	273	271	261	248	241	250	275	272	276	273	268	262	253	246
N70 trancript lenght (bp)	921	553	582	584	557	516	465	464	531	454	485	472	450	427	405	386
N50 trancript lenght (bp)	1964	1610	1343	1361	1321	1256	1117	1107	1234	755	854	842	808	765	723	682
N30 trancript lenght (bp)	3346	3262	2852	2896	2843	2741	2507	2396	2496	1288	1517	1537	1505	1463	1408	1352
N10 trancript lenght (bp)	6008	6159	5886	5985	5934	5729	5279	5014	5014	2592	3140	3320	3272	3222	3107	3031
Percentage of GC (%)	0.44	0.43	0.43	0.43	0.43	0.43	0.43	0.43	0.44	0.43	0.43	0.43	0.43	0.43	0.43	0.43
**Busco analysis (%)**	—															
BUSCO Complete (Single + Duplicated)*	97.03\97.34\80.78\72.45	92.74\93.35\73.05\64.18	97.03\96.22\78.96\70.64	96.37\96.01\78.23\70.88	96.04\95.60\77.49\70.07	95.05\94.99\77.30\68.59	94.06\93.87\72.89\65.34	91.09\92.33\92.33\64.11	91.42\90.80\68.64\61.21	63.04\65.13\38.86\31.22	63.04\66.67\40.56\34.23	64.36\66.56\40.64\34.21	64.03\69.63\42.38\35.73	69.97\74.13\44.97\37.85	72.94\77.10\47.72\40.29	76.90\79.14\49.57\41.97
BUSCO Single*	13.20\13.60\16.36\14.83	64.03\68.81\56.84\50.76	87.79\87.83\72.89\64.59	88.45\87.42\72.39\64.53	87.46\87.12\71.58\63.70	85.15\86.09\71.50\63.11	83.83\84.46\67.05\59.53	81.52\83.23\83.23\58.53	81.52\79.65\62.57\55.54	61.39\63.39\38.21\30.85	61.06\65.75\39.79\33.73	61.72\65.24\39.95\33.46	62.38\67.59\41.57\34.95	68.98\71.98\44.12\37.00	71.62\74.95\46.71\39.42	75.25\76.99\48.53\41.03
BUSCO Duplicated*	83.83\83.74\64.42\57.61	28.71\24.54\16.20\13.42	9.24\8.38\6.07\6.04	7.92\8.59\5.84\6.35	8.58\8.49\5.92\6.37	9.90\8.90\5.80\5.48	10.23\9.41\5.84\5.80	9.57\9.10\9.10\5.58	9.90\11.15\6.07\5.67	1.65\1.74\0.66\0.37	1.98\0.92\0.77\0.50	2.64\1.33\0.70\0.74	1.65\2.04\0.81\0.79	0.99\2.15\0.85\0.85	1.32\2.15\1.01\0.87	1.65\2.15\1.04\0.94
BUSCO Fragmented*	1.98\1.94\8.51\7.44	6.60\5.93\16.59\13.90	1.98\2.76\10.60\9.05	2.31\3.07\11.06\8.64	2.97\3.37\11.52\8.99	3.96\4.19\11.25\9.90	5.28\4.81\14.54\11.71	7.59\6.24\6.24\11.87	6.93\7.67\17.13\13.59	33.66\31.19\43.35\29.97	34.32\30.27\41.80\28.82	32.67\30.06\41.69\29.08	34.98\27.61\39.87\28.69	28.71\23.72\37.39\27.55	25.08\20.86\35.23\26.18	21.45\19.12\33.57\24.65
BUSCO Missing*	0.99\0.72\10.71\20.11	0.66\0.72\10.36\21.92	0.99\1.02\10.44\20.31	1.32\0.92\10.71\20.48	0.99\1.02\10.98\20.94	0.99\0.82\11.45\21.51	0.66\1.33\12.57\22.95	1.32\1.43\1.43\24.02	1.65\1.53\14.23\25.20	3.30\3.68\17.79\38.81	2.64\3.07\17.63\36.95	2.97\3.37\17.67\36.71	0.99\2.76\17.75\35.58	1.32\2.15\17.63\34.60	1.98\2.04\17.05\33.53	1.65\1.74\16.86\33.38
Total Buscos Found*	99.01\99.28\89.29\79.89	99.34\99.28\89.64\78.08	99.01\98.98\89.56\79.69	98.68\99.08\89.29\79.52	99.01\98.98\89.02\79.06	99.01\99.18\88.55\78.49	99.34\98.67\87.43\77.05	98.68\98.57\98.57\75.98	98.35\98.47\85.77\74.80	96.70\96.32\82.21\61.19	97.36\96.93\82.37\63.05	97.03\96.63\82.33\63.29	99.01\97.24\82.25\64.42	98.68\97.85\82.37\65.40	98.02\97.96\82.95\66.47	98.35\98.26\83.14\66.62

*euk/met/ver/act

Euk: Dataset with 303 genes of Eukaryota library profile.

Met: Dataset with 978 genes of Metazoa library profile.

Ver: Dataset with 2586 genes of Vertebrata library profile

Actino: Dataset with 4584 genes of Actinopterygii library profile.

**Online-only Table 3:** Transrate and Busco statistics of the 16 initial liver transcriptome assemblies of T. luscus.

Basic statistics	Trinity	RnaSpades	SOAPdenovo-Trans (k25)	SOAPdenovo-Trans (k31)	SOAPdenovo-Trans (k41)	SOAPdenovo-Trans (k51)	SOAPdenovo-Trans (k61)	SOAPdenovo-Trans (k71)	SOAPdenovo-Trans (k81)	Idba-trans (k25)	Idba-trans (k31)	Idba-trans (k41)	Idba-trans (k51)	Idba-trans (k61)	Idba-trans (k71)	Idba-trans (k81)
Number of transcripts	411058	292811	154794	160812	169625	178939	188647	181540	153558	179390	182171	185489	189073	195450	201382	204619
Longest transcript	32751	23400	19450	17026	17906	21246	19563	19466	17065	13829	13875	13939	13807	14323	15162	13521
n bases	320646510	200542845	98646712	104201244	107378705	107861212	107809770	104007203	95013750	88082035	95851297	99609397	100437843	100142391	98964534	96881871
Mean transcript lenght (bp)	780.05	684.89	637.28	647.97	633.04	602.78	571.49	572.92	618.75	491.01	526.16	537.01	531.21	512.37	491.43	473.47
Number of transcripts over 1 K nt	84119	46332	22508	23557	23788	23364	22835	22274	21686	14387	17980	19449	19474	18632	17564	16470
Number of transcripts over 10 K nt	250	117	57	74	90	77	67	56	44	2	5	8	9	7	9	10
N90 trancript lenght (bp)	321	278	259	260	253	244	238	244	265	256	262	263	260	251	242	235
N70 trancript lenght (bp)	615	485	478	484	464	429	397	393	432	371	396	401	394	375	356	340
N50 trancript lenght (bp)	1231	1102	958	994	973	912	837	824	907	555	615	638	632	598	561	529
N30 trancript lenght (bp)	2217	2263	1953	2076	2055	1972	1839	1786	1849	884	1024	1091	1107	1079	1029	990
N10 trancript lenght (bp)	4245	4492	4059	4300	4367	4187	3961	3834	3711	1725	2049	2226	2320	2313	2260	2228
Percentage of GC (%)	0.53	0.52	0.51	0.51	0.51	0.51	0.51	0.52	0.52	0.5	0.51	0.51	0.51	0.51	0.51	0.52
**Busco analysis (%)**	—															
BUSCO Complete (Single + Duplicated)*	100.00\98.57\82.56\74.45	95.38\96.42\77.53\70.51	98.35\97.55\80.51\71.10	98.68\97.75\81.55\73.39	99.01\97.65\81.52\73.67	97.36\96.73\80.01\72.36	96.70\94.68\75.14\68.26	93.73\93.25\72.66\64.75	92.08\92.84\69.68\62.15	58.09\64.21\35.69\27.07	66.34\72.90\41.65\34.27	70.63\78.22\44.74\38.11	71.95\79.14\49.19\42.30	77.23\82.21\51.82\44.20	80.86\84.15\53.79\46.42	84.49\85.28\56.26\47.77
BUSCO Single*	7.26\7.77\11.52\11.65	66.34\67.69\58.20\52.33	92.74\91.62\75.95\66.45	92.41\90.80\76.57\68.02	92.08\89.78\76.95\68.19	89.44\88.75\75.37\66.84	86.80\86.40\69.61\62.33	81.19\82.11\65.85\58.57	75.58\78.63\60.98\54.30	56.44\62.88\35.11\26.79	64.36\71.27\40.87\33.57	68.98\76.58\43.97\37.30	70.30\77.71\48.34\41.34	75.25\80.06\50.85\43.28	79.21\81.39\52.71\45.29	82.84\81.70\55.22\46.58
BUSCO Duplicated*	92.74\90.80\71.04\62.81	29.04\28.73\19.33\18.17	5.61\5.93\4.56\4.65	6.27\6.95\4.99\5.37	6.93\7.87\4.56\5.48	7.92\7.98\4.64\5.52	9.90\8.28\5.53\5.93	12.54\11.15\6.81\6.17	16.50\14.21\8.70\7.85	1.65\1.33\0.58\0.28	1.98\1.64\0.77\0.70	1.65\1.64\0.77\0.81	1.65\1.43\0.85\0.96	1.98\2.15\0.97\0.92	1.65\2.76\1.08\1.13	1.65\3.58\1.04\1.20
BUSCO Fragmented*	0.00\1.23\9.78\8.94	4.29\3.37\14.97\12.22	1.32\2.25\11.21\10.51	1.32\1.94\10.67\9.77	0.99\2.04\10.56\9.27	2.31\2.86\11.52\10.25	3.30\4.91\15.74\12.67	6.27\6.13\17.32\14.73	7.92\6.54\19.84\15.34	37.62\31.49\46.98\31.06	32.34\24.54\43.74\30.28	29.04\20.45\40.99\29.19	28.05\19.84\37.59\26.96	22.44\17.28\35.58\26.13	19.14\15.54\33.80\25.00	15.51\14.31\31.28\24.17
BUSCO Missing*	0.00\0.20\7.66\16.60	0.33\0.20\7.50\17.28	0.33\0.20\8.28\18.39	0.00\0.31\7.77\16.84	0.00\0.31\7.93\17.06	0.33\0.41\8.47\17.39	0.00\0.41\9.13\19.07	0.00\0.61\10.02\20.53	0.00\0.61\10.48\22.51	4.29\4.29\17.32\41.86	1.32\2.56\14.62\35.45	0.33\1.33\14.27\32.70	0.00\1.02\13.23\30.74	0.33\0.51\12.61\29.67	0.00\0.31\12.41\28.58	0.00\0.41\12.45\28.05
Total Buscos Found*	100.00\99.80\92.34\83.40	99.67\99.80\92.50\82.72	99.67\99.80\91.72\81.61	100.00\99.69\92.23\83.16	100.00\99.69\92.07\82.94	99.67\99.59\91.53\82.61	100.00\99.59\90.87\80.93	100.00\99.39\89.98\79.47	100.00\99.39\89.52\77.49	95.71\95.71\82.68\58.14	98.68\97.44\85.38\64.55	99.67\98.67\85.73\67.30	100.00\98.98\86.77\69.26	99.67\99.49\87.39\70.33	100.00\99.69\87.59\71.42	100.00\99.59\87.55\71.95

*euk/met / ver/act

Euk: Dataset with 303 genes of Eukaryota library profile.

Met: Dataset with 978 genes of Metazoa library profile.

Ver: Dataset with 2586 genes of Vertebrata library profile

Actino: Dataset with 4584 genes of Actinopterygii library profile.

**Online-only Table 4:** Transrate and Busco statistics of the 16 initial liver transcriptome assemblies of M. poutassou.

Basic statistics	Trinity	RnaSpades	SOAPdenovo-Trans (k25)	SOAPdenovo-Trans (k31)	SOAPdenovo-Trans (k41)	SOAPdenovo-Trans (k51)	SOAPdenovo-Trans (k61)	SOAPdenovo-Trans (k71)	SOAPdenovo-Trans (k81)	Idba-trans (k25)	Idba-trans (k31)	Idba-trans (k41)	Idba-trans (k51)	Idba-trans (k61)	Idba-trans (k71)	Idba-trans (k81)
Number of transcripts	464167	330050	177545	186370	195930	207329	216930	206477	172974	208443	210941	215238	220501	226879	231007	232815
Longest transcript	21943	24472	19591	18778	21129	21290	19702	26373	19501	14207	14848	13517	14471	14052	14191	15424
n bases	362993700	233843506	117331079	123189353	127788108	128934716	128494411	123468531	112004054	103493945	111919073	116653625	118143740	117849403	116075407	112981783
Mean transcript lenght (bp)	782.03	708.51	660.85	660.99	652.21	621.88	592.33	597.98	647.52	496.51	530.57	541.98	535.8	519.44	502.48	485.29
Number of transcripts over 1 K nt	93608	55128	26856	27659	28223	27820	26929	26060	25078	17044	21013	22733	22718	21742	20655	19261
Number of transcripts over 10 K nt	237	165	84	97	115	111	102	83	70	4	9	12	18	15	18	19
N90 trancript lenght (bp)	322	279	265	265	257	247	242	248	270	258	264	266	262	256	247	239
N70 trancript lenght (bp)	614	512	502	499	487	451	420	417	457	377	401	407	400	384	368	352
N50 trancript lenght (bp)	1228	1193	1013	1003	1009	950	877	881	968	563	621	642	635	607	578	548
N30 trancript lenght (bp)	2261	2387	2079	2101	2158	2090	1965	1956	2032	892	1022	1089	1100	1069	1041	1007
N10 trancript lenght (bp)	4313	4649	4363	4482	4655	4520	4280	4259	4251	1731	2050	2258	2317	2323	2320	2320
Percentage of GC (%)	0.51	0.5	0.49	0.49	0.49	0.49	0.5	0.5	0.51	0.48	0.49	0.49	0.49	0.49	0.5	0.5
**Busco analysis (%)**	—															
BUSCO Complete (Single + Duplicated)*	100.00\98.88\83.64\75.28	94.39\95.71\77.34\69.87	98.35\97.34\82.13\72.40	99.34\97.96\82.87\74.39	99.01\97.44\83.06\75.15	97.36\96.83\80.74\73.91	96.37\95.40\77.03\70.09	93.40\94.58\75.33\68.41	93.40\93.66\74.25\66.36	66.67\65.54\33.02\24.80	72.94\71.06\38.01\30.37	73.27\71.98\39.40\32.70	75.91\72.80\41.07\36.32	76.24\74.85\44.04\38.57	79.87\78.63\48.26\42.19	83.50\83.03\83.03\44.55
BUSCO Single*	8.25\8.18\13.11\12.70	58.75\65.24\57.42\50.48	86.47\87.63\76.26\66.51	87.79\88.34\76.49\68.22	85.48\87.63\76.57\68.35	83.17\86.30\73.98\66.84	81.19\83.33\70.07\62.54	78.88\81.90\67.79\60.43	77.56\79.86\66.82\58.12	64.69\62.68\32.52\24.24	69.31\68.00\37.28\29.82	69.64\68.20\38.32\31.61	71.95\68.30\39.98\34.95	71.62\69.43\43.12\37.00	76.24\73.21\47.02\40.36	78.22\76.69\76.69\42.54
BUSCO Duplicated*	91.75\90.70\70.53\62.59	35.64\30.47\19.91\19.39	11.88\9.71\5.88\5.89	11.55\9.61\6.38\6.17	13.53\9.82\6.50\6.81	14.19\10.53\6.77\7.07	15.18\12.07\6.96\7.55	14.52\12.68\7.54\7.98	15.84\13.80\7.42\8.25	1.98\2.86\0.50\0.57	3.63\3.07\0.73\0.55	3.63\3.78\1.08\1.09	3.96\4.50\1.08\1.37	4.62\5.42\0.93\1.57	3.63\5.42\1.24\1.83	5.28\6.34\6.34\2.01
BUSCO Fragmented*	0.00\0.82\8.78\8.70	5.28\4.09\15.74\12.65	1.32\2.25\10.56\9.51	0.66\1.64\10.05\8.94	0.99\2.15\9.67\8.60	2.31\2.76\11.18\9.14	3.30\3.99\14.54\11.23	5.94\4.91\15.70\12.65	6.60\5.52\15.58\13.26	31.68\30.67\47.18\30.63	26.07\26.79\45.90\30.58	25.74\25.66\45.24\31.46	23.43\25.46\44.43\29.43	23.10\24.03\42.23\29.30	19.80\20.45\38.55\27.25	16.50\16.26\16.26\26.00
BUSCO Missing*	0.00\0.31\7.58\16.01	0.33\0.20\6.92\17.47	0.33\0.41\7.31\18.08	0.00\0.41\7.08\16.67	0.00\0.41\7.27\16.25	0.33\0.41\8.08\16.95	0.33\0.61\8.43\18.67	0.66\0.51\8.97\18.94	0.00\0.82\10.17\20.38	1.65\3.78\19.80\44.57	0.99\2.15\16.09\39.05	0.99\2.35\15.35\35.84	0.66\1.74\14.50\34.25	0.66\1.12\13.73\32.13	0.33\0.92\13.19\30.56	0.00\0.72\0.72\29.45
Total Buscos Found*	100.00\99.69\92.42\83.99	99.67\99.80\93.08\82.53	99.67\99.59\92.69\81.92	100.00\99.59\92.92\83.33	100.00\99.59\92.73\83.75	99.67\99.59\91.92\83.05	99.67\99.39\91.57\81.33	99.34\99.49\91.03\81.06	100.00\99.18\89.83\79.62	98.35\96.22\80.20\55.43	99.01\97.85\83.91\60.95	99.01\97.65\84.65\64.16	99.34\98.26\85.50\65.75	99.34\98.88\86.27\67.87	99.67\99.08\86.81\69.44	100.00\99.28\99.28\70.55

*euk/met/ver/act

Euk: Dataset with 303 genes of Eukaryota library profile.

Met: Dataset with 978 genes of Metazoa library profile.

Ver: Dataset with 2586 genes of Vertebrata library profile

Actino: Dataset with 4584 genes of Actinopterygii library profile.

**Online-only Table 5:** Transrate, Busco and RBMT statistics of multi and non-redudant liver transcriptome assemblies of *T. trachurus, S. scombrus, T. luscus, M. poutassou*.

Basic statistics	Multi-assembly (*T. trachurus*)	Non-redundat assembly (*T. trachurus*)	Multi-assembly (*S. scombrus*)	Non-redundat assembly (*S. scombrus*)	Multi-assembly (*T. luscus*)	Non-redundat assembly (*T. luscus*)	Multi-assembly (*M. poutassou*)	Non-redundat assembly (*M. poutassou*)
Number of transcripts	2769441	414729	2728965	377586	3203445	548983	3675167	602418
Longest transcript	29599	29599	44312	44312	32751	32751	26373	26373
n bases	1747321952	368691712	1890204885	380142640	1907624448	430348125	2233549078	494330249
Mean transcript lenght (bp)	630.93	888.99	692.65	1006.77	595.49	783.9	607.74	820.58
Number of transcripts over 1 K nt	394404	95958	431868	93637	409605	108225	476782	125499
Number of transcripts over 10 K nt	1190	574	2104	1072	871	384	1144	535
N90 trancript lenght (bp)	261	294	272	324	262	289	265	294
N70 trancript lenght (bp)	463	819	522	1015	430	630	442	681
N50 trancript lenght (bp)	937	1825	1121	2324	800	1396	818	1512
N30 trancript lenght (bp)	1898	3181	2342	4014	1622	2606	1666	2793
N10 trancript lenght (bp)	4166	5934	4998	7110	3568	4986	3755	5273
Percentage of GC (%)	0.46	0.47	0.43	0.44	0.52	0.53	0.5	0.51
Rate mapping %	—	96.78	—	97.16	—	95.3	—	95.51
**Busco analysis (%)**	—							
BUSCO Complete (Single + Duplicated)*	98.02\97.03\82.13\76.70	99.34\98.26\84.11\78.45	97.03\97.14\82.68\76.03	98.02\97.85\84.61\76.96	99.67\97.85\85.11\79.67	99.67\99.08\88.28\82.02	100.00\97.96\86.23\80.54	99.67\99.39\89.13\82.57
BUSCO Single*	2.97\2.15\3.48\3.97	35.97\34.05\32.68\30.52	1.98\2.25\3.40\4.65	32.34\32.21\33.49\31.76	1.98\2.76\3.52\4.62	24.09\28.32\30.12\28.47	1.32\1.94\3.48\4.45	23.76\27.71\28.38\28.34
BUSCO Duplicated*	95.05\94.89\78.65\72.73	63.37\64.21\51.43\47.93	95.05\94.89\79.27\71.38	65.68\65.64\51.12\45.20	97.69\95.09\81.59\75.04	75.58\70.76\58.16\53.56	98.68\96.01\82.75\76.09	75.91\71.68\60.75\54.23
BUSCO Fragmented*	1.98\2.56\9.63\7.66	0.66\1.33\7.77\6.22	2.31\2.25\8.39\6.15	1.32\1.53\6.54\5.24	0.00\1.84\8.47\6.68	0.00\0.61\5.34\4.47	0.00\1.84\7.70\5.93	0.33\0.41\4.80\4.19
BUSCO Missing*	0.00\0.41\8.24\15.64	0.00\0.41\8.12\15.34	0.66\0.61\8.93\17.82	0.66\0.61\8.86\17.80	0.33\0.31\6.42\13.66	0.33\0.31\6.38\13.50	0.00\0.20\6.07\13.53	0.00\0.20\6.07\13.24
Total Buscos Found*	100.00\99.59\91.76\84.36	100.00\99.59\91.88\84.66	99.34\99.39\91.07\82.18	99.34\99.39\91.14\82.20	99.67\99.69\93.58\86.34	99.67\99.69\93.62\86.50	100.00\99.80\93.93\86.47	100.00\99.80\93.93\86.76

*euk/met/ver/act

Euk: Dataset with 303 genes of Eukaryota library profile.

Met: Dataset with 978 genes of Metazoa library profile.

Ver: Dataset with 2586 genes of Vertebrata library profile

Actino: Dataset with 4584 genes of Actinopterygii library profile.
